# *N*-[4-(Di­methyl­amino)­benzyl­idene]-4-methyl­aniline

**DOI:** 10.1107/S160053681301249X

**Published:** 2013-05-18

**Authors:** R. K. Balachandar, S. Kalainathan, Shibu M Eappen, Jiban Podder

**Affiliations:** a Centre for Crystal Growth, School of Advanced Sciences, VIT University, Vellore 632 014, India; b Sophisticated Test and Instrumentation Centre (STIC), Cochin University PO, Cochin 682 022, Kerala; c Department of Physics, Bangladesh University of Engineering and Technology, Dhaka 1000, Bangladesh

## Abstract

The mol­ecules of the title compound, C_16_H_18_N_2_, exists in a *trans* conformation with respect to the C=N bond [1.270 (3) Å]. The least-squares plane of the di­methyl­amino group makes a dihedral angle of 1.3 (2)° with the ring to which it is attached. The dihedral angle between the two aromatic rings is 11.70 (2)°. The crystal structure features weak C—H⋯π inter­actions.

## Related literature

For the uses and biological importance of diketones, see: Xia *et al.* (2009 ▶); Shah *et al.* (1992 ▶); Ünver *et al.* (2004 ▶). For related structures, see: Fun *et al.* (2011 ▶); Khalaji & Simpson (2009 ▶).
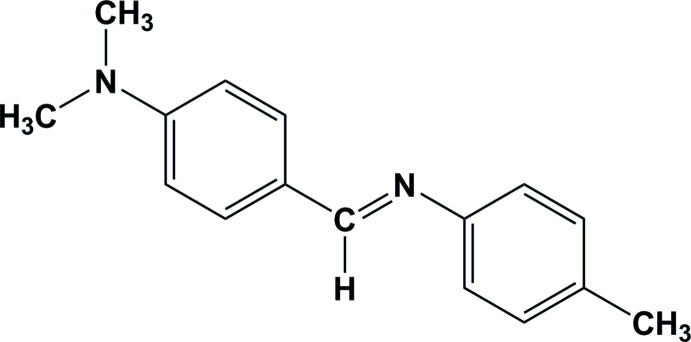


## Experimental

### 

#### Crystal data

C_16_H_18_N_2_
*M* *_r_* = 238.32
Orthorhombic, 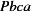

*a* = 10.4814 (10) Å
*b* = 8.0528 (8) Å
*c* = 32.571 (3) Å
*V* = 2749.1 (4) Å^3^
*Z* = 8
Mo *K*α radiation
μ = 0.07 mm^−1^
*T* = 296 K
0.30 × 0.25 × 0.20 mm

#### Data collection

Bruker Kappa APEXII CCD diffractometer
Absorption correction: multi-scan (*SADABS*; Bruker, 2004 ▶) *T*_min_ = 0.980, *T*_max_ = 0.987
17121 measured reflections
2692 independent reflections
1737 reflections with *I* > 2σ(*I*)
*R*_int_ = 0.041

#### Refinement

*R*[*F* ^2^ > 2σ(*F* ^2^)] = 0.061
*wR*(*F* ^2^) = 0.187
*S* = 1.08
2692 reflections
167 parameters
H-atom parameters constrained
Δρ_max_ = 0.21 e Å^−3^
Δρ_min_ = −0.16 e Å^−3^

### 

Data collection: *APEX2* (Bruker, 2004 ▶); cell refinement: *APEX2* and *SAINT* (Bruker, 2004 ▶); data reduction: *SAINT* and *XPREP* (Bruker, 2004 ▶); program(s) used to solve structure: *SIR92* (Altomare *et al.*, 1993 ▶); program(s) used to refine structure: *SHELXL97* (Sheldrick, 2008 ▶); molecular graphics: *ORTEP-3 for Windows* (Farrugia, 2012 ▶); software used to prepare material for publication: *SHELXL97* and *PLATON* (Spek, 2009 ▶).

## Supplementary Material

Crystal structure: contains datablock(s) global, I. DOI: 10.1107/S160053681301249X/bt6904sup1.cif

Structure factors: contains datablock(s) I. DOI: 10.1107/S160053681301249X/bt6904Isup2.hkl

Supplementary material file. DOI: 10.1107/S160053681301249X/bt6904Isup3.cml

Additional supplementary materials:  crystallographic information; 3D view; checkCIF report

## Figures and Tables

**Table 1:** Hydrogen-bond geometry (Å, °)

*D*—H⋯*A*	*D*—H	H⋯*A*	*D*⋯*A*	*D*—H⋯*A*
C11—H11⋯*Cg*1^i^	0.93	2.94	3.670 (2)	137

Symmetry code: (i) 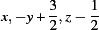.

**Fig. 1.**
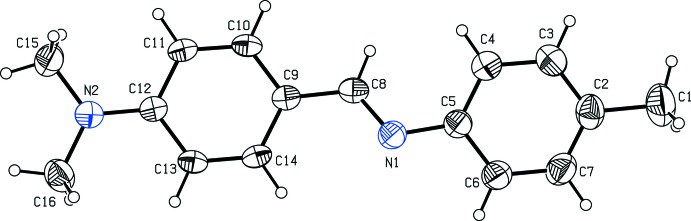
The molecular structure of the title compound with the atom numbering scheme, displacement ellipaoids are drawn at 30% probability level. H atoms are present as small spheres of arbitary radius.

**Fig. 2.**
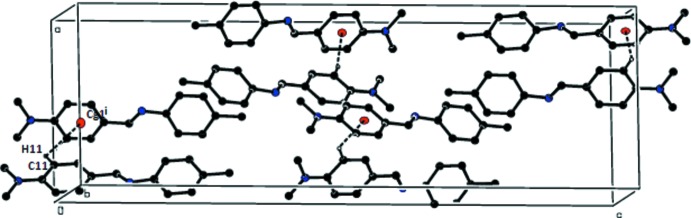
The crystal packing of the title compound, viewed down a-axis, showing C11—H11···Cg1i inter-molecular interactions. The H atoms not involved in the bonding have been excluded for clarity.

## supplementary crystallographic information

### Comment

Schiff bases are among the most useful ligands in coordination chemistry as they readily form stable complexes with most transition metals (Xia *et al.*, 2009). They are known to exibit potent anti-bacterial, anti-convulsant, anti-inflammatory and anti-cancer activities (Shah *et al.*, 1992). In addition to that, they show Non-linear optical properties (Ünver *et al.*, 2004). Therefore, successful application of Schiff bases requires a careful study of their characteristics.

The title compound, C_16_H_18_N_2_, exists in a *trans* configuration with respect to the C═N bond[1.270 (3) Å]. The N1═C8 bond length of 1.270 (3) Å is shorter than the N–C bond [1.413 (3) Å], indicating a typical imine double bond. The C–N–C angle is 120.6 (2) °. X-ray analysis confirms the molecular structure and atom connectivity as illustrated in Fig. 1.

The least square plane of the dimethylamine group has a dihedral angle of 1.31 (2) ° with the phenyl ring (C9–C14), which shows that they are almost coplanar to each other. The dimethylamine group attached phenyl ring (C9–C14) forms a dihedral angle of 11.70 (2) Å with the methyl group attached phenyl ring (C2–C7).

The crystal packing is stabilized by C11—H11···*Cg*1^i^ inter-molecular interactions, where *Cg*1 is the center of gravity of (C8–C14) phenyl ring. The symmetry code is 1/2-*X*,-1/2+Y,*Z*.

### Experimental

The title compound was synthesized by the reaction of *p*-dimethylaminobenzaldehyde (10 mmol, 1.14919 g) with p-toluidine (10 mmol, 1.0717 g) in ethanol (25 ml) under reflux condition for six hours. After filtering, drying the solid product was recrystallized from ethanol/THF (5:1 *v*/*v*). After five days yellow colour crystals were obtained Which were suitable for X-ray diffraction studies.

### Refinement

The positions of hydrogen atoms were localized from the difference electron density maps and their distances were geometrically constrained. The H atoms bound to the C atoms were treated as riding atoms, with d(C–H) = 0.93 Å and *U*_iso_(H) = 1.2*U*_eq_(C) for aryl atoms; d(C–H) = 0.96 Å and *U*_iso_(H) = 1.5*U*_eq_(C) for methyl group. The methyl groups were allowed to rotate but not to tip.

### Crystal data

**Table:**

C_16_H_18_N_2_	*F*(000) = 1024
*M**_r_* = 238.32	*D*_x_ = 1.152 Mg m^−^^3^
Orthorhombic, *P**b**c**a*	Mo *K*α radiation, λ = 0.71073 Å
Hall symbol: -P 2ac 2ab	Cell parameters from 1737 reflections
*a* = 10.4814 (10) Å	θ = 2.3–26.0°
*b* = 8.0528 (8) Å	µ = 0.07 mm^−^^1^
*c* = 32.571 (3) Å	*T* = 296 K
*V* = 2749.1 (4) Å^3^	Block, yellow
*Z* = 8	0.30 × 0.25 × 0.20 mm

### Data collection

**Table:**

Bruker Kappa APEXII CCD diffractometer	2692 independent reflections
Radiation source: fine-focus sealed tube	1737 reflections with *I* > 2σ(*I*)
Graphite monochromator	*R*_int_ = 0.041
ω and φ scan	θ_max_ = 26.0°, θ_min_ = 2.3°
Absorption correction: multi-scan (*SADABS*; Bruker, 2004)	*h* = −12→12
*T*_min_ = 0.980, *T*_max_ = 0.987	*k* = −9→7
17121 measured reflections	*l* = −40→40

### Refinement

**Table:**

Refinement on *F*^2^	Secondary atom site location: difference Fourier map
Least-squares matrix: full	Hydrogen site location: inferred from neighbouring sites
*R*[*F*^2^ > 2σ(*F*^2^)] = 0.061	H-atom parameters constrained
*wR*(*F*^2^) = 0.187	*w* = 1/[σ^2^(*F*_o_^2^) + (0.0814*P*)^2^ + 0.8121*P*] where *P* = (*F*_o_^2^ + 2*F*_c_^2^)/3
*S* = 1.08	(Δ/σ)_max_ = 0.001
2692 reflections	Δρ_max_ = 0.21 e Å^−^^3^
167 parameters	Δρ_min_ = −0.16 e Å^−^^3^
0 restraints	Extinction correction: *SHELXL97* (Sheldrick, 2008), Fc^*^=kFc[1+0.001xFc^2^λ^3^/sin(2θ)]^-1/4^
Primary atom site location: structure-invariant direct methods	Extinction coefficient: 0.0130 (19)

### Special details

**Table:**

Geometry. All e.s.d.'s (except the e.s.d. in the dihedral angle between two l.s. planes) are estimated using the full covariance matrix. The cell e.s.d.'s are taken into account individually in the estimation of e.s.d.'s in distances, angles and torsion angles; correlations between e.s.d.'s in cell parameters are only used when they are defined by crystal symmetry. An approximate (isotropic) treatment of cell e.s.d.'s is used for estimating e.s.d.'s involving l.s. planes.
Refinement. Refinement of *F*^2^ against ALL reflections. The weighted *R*-factor *wR* and goodness of fit *S* are based on *F*^2^, conventional *R*-factors *R* are based on *F*, with *F* set to zero for negative *F*^2^. The threshold expression of *F*^2^ > σ(*F*^2^) is used only for calculating *R*-factors(gt) *etc*. and is not relevant to the choice of reflections for refinement. *R*-factors based on *F*^2^ are statistically about twice as large as those based on *F*, and *R*- factors based on ALL data will be even larger.

### Fractional atomic coordinates and isotropic or equivalent isotropic displacement parameters (Å^2^)

**Table:**

	*x*	*y*	*z*	*U*_iso_*/*U*_eq_	
C6	0.0030 (3)	0.4888 (3)	0.18005 (8)	0.0768 (8)	
H6	−0.0658	0.5293	0.1651	0.092*	
C14	0.0373 (2)	0.2889 (2)	0.03147 (7)	0.0523 (6)	
H14	−0.0182	0.3671	0.0425	0.063*	
C13	0.0307 (2)	0.2534 (3)	−0.00941 (7)	0.0528 (6)	
H13	−0.0297	0.3073	−0.0255	0.063*	
C15	0.1898 (3)	−0.0172 (4)	−0.08750 (8)	0.0805 (8)	
H15A	0.2757	0.0243	−0.0875	0.121*	
H15B	0.1629	−0.0378	−0.1152	0.121*	
H15C	0.1862	−0.1187	−0.0721	0.121*	
C16	0.0139 (3)	0.1841 (4)	−0.09499 (8)	0.0809 (8)	
H16A	−0.0705	0.1568	−0.0857	0.121*	
H16B	0.0247	0.1468	−0.1228	0.121*	
H16C	0.0258	0.3022	−0.0938	0.121*	
C10	0.2053 (2)	0.0940 (2)	0.03889 (7)	0.0530 (6)	
H10	0.2640	0.0380	0.0552	0.064*	
C1	0.1192 (4)	0.5064 (5)	0.29002 (9)	0.1148 (13)	
H1A	0.1367	0.6223	0.2939	0.172*	
H1B	0.1863	0.4416	0.3021	0.172*	
H1C	0.0395	0.4787	0.3029	0.172*	
C3	0.2043 (3)	0.3782 (4)	0.22509 (9)	0.0862 (9)	
H3	0.2737	0.3400	0.2402	0.103*	
C7	0.0115 (3)	0.5248 (4)	0.22110 (9)	0.0831 (9)	
H7	−0.0521	0.5886	0.2333	0.100*	
C4	0.1978 (3)	0.3420 (4)	0.18370 (8)	0.0788 (8)	
H4	0.2632	0.2823	0.1713	0.095*	
C2	0.1112 (3)	0.4694 (3)	0.24474 (8)	0.0791 (8)	
C9	0.1257 (2)	0.2105 (2)	0.05712 (7)	0.0482 (5)	
C12	0.11276 (19)	0.1375 (2)	−0.02776 (7)	0.0477 (5)	
C5	0.0941 (2)	0.3941 (3)	0.16042 (7)	0.0607 (6)	
N2	0.10664 (19)	0.1037 (2)	−0.06899 (6)	0.0632 (6)	
N1	0.0775 (2)	0.3645 (2)	0.11798 (6)	0.0630 (6)	
C11	0.2007 (2)	0.0582 (2)	−0.00217 (7)	0.0527 (6)	
H11	0.2567	−0.0197	−0.0131	0.063*	
C8	0.1356 (2)	0.2458 (3)	0.10037 (7)	0.0541 (6)	
H22E	0.1874	0.1778	0.1163	0.065*	

### Atomic displacement parameters (Å^2^)

**Table:**

	*U*^11^	*U*^22^	*U*^33^	*U*^12^	*U*^13^	*U*^23^
C6	0.0832 (19)	0.0809 (18)	0.0664 (17)	0.0181 (15)	0.0077 (14)	0.0081 (14)
C14	0.0464 (12)	0.0402 (11)	0.0703 (15)	0.0053 (9)	0.0058 (11)	0.0021 (10)
C13	0.0470 (13)	0.0445 (11)	0.0669 (14)	0.0029 (9)	−0.0055 (11)	0.0087 (10)
C15	0.0811 (19)	0.0882 (19)	0.0721 (16)	0.0062 (15)	0.0070 (15)	−0.0134 (14)
C16	0.099 (2)	0.0781 (18)	0.0657 (16)	0.0016 (15)	−0.0108 (15)	0.0086 (13)
C10	0.0463 (13)	0.0431 (11)	0.0696 (14)	0.0046 (9)	−0.0046 (11)	0.0095 (10)
C1	0.147 (3)	0.132 (3)	0.0655 (18)	−0.002 (3)	0.001 (2)	−0.0060 (18)
C3	0.094 (2)	0.091 (2)	0.0741 (18)	0.0121 (17)	−0.0165 (16)	−0.0009 (15)
C7	0.092 (2)	0.085 (2)	0.0722 (18)	0.0140 (16)	0.0183 (17)	0.0006 (15)
C4	0.077 (2)	0.0833 (19)	0.0759 (17)	0.0150 (15)	−0.0038 (15)	−0.0099 (14)
C2	0.103 (2)	0.0753 (18)	0.0593 (16)	−0.0054 (16)	0.0072 (16)	0.0052 (13)
C9	0.0459 (12)	0.0381 (11)	0.0607 (13)	−0.0047 (8)	0.0043 (10)	0.0078 (9)
C12	0.0422 (12)	0.0411 (11)	0.0597 (13)	−0.0066 (8)	0.0038 (10)	0.0035 (9)
C5	0.0697 (17)	0.0530 (13)	0.0592 (14)	0.0018 (11)	0.0053 (12)	0.0076 (11)
N2	0.0613 (13)	0.0650 (12)	0.0632 (12)	0.0064 (10)	−0.0013 (10)	0.0009 (10)
N1	0.0684 (14)	0.0608 (12)	0.0599 (12)	0.0050 (10)	0.0046 (10)	0.0042 (9)
C11	0.0443 (13)	0.0429 (11)	0.0710 (14)	0.0051 (9)	0.0024 (11)	0.0004 (10)
C8	0.0524 (13)	0.0428 (12)	0.0670 (14)	−0.0018 (9)	0.0016 (11)	0.0110 (10)

### Geometric parameters (Å, º)

**Table:**

C6—C7	1.371 (4)	C1—C2	1.507 (4)
C6—C5	1.378 (3)	C1—H1A	0.9600
C6—H6	0.9300	C1—H1B	0.9600
C14—C13	1.364 (3)	C1—H1C	0.9600
C14—C9	1.398 (3)	C3—C2	1.379 (4)
C14—H14	0.9300	C3—C4	1.381 (4)
C13—C12	1.403 (3)	C3—H3	0.9300
C13—H13	0.9300	C7—C2	1.373 (4)
C15—N2	1.439 (3)	C7—H7	0.9300
C15—H15A	0.9600	C4—C5	1.390 (3)
C15—H15B	0.9600	C4—H4	0.9300
C15—H15C	0.9600	C9—C8	1.441 (3)
C16—N2	1.443 (3)	C12—N2	1.372 (3)
C16—H16A	0.9600	C12—C11	1.397 (3)
C16—H16B	0.9600	C5—N1	1.413 (3)
C16—H16C	0.9600	N1—C8	1.270 (3)
C10—C11	1.369 (3)	C11—H11	0.9300
C10—C9	1.389 (3)	C8—H22E	0.9300
C10—H10	0.9300		
			
C7—C6—C5	121.6 (3)	C2—C3—H3	119.0
C7—C6—H6	119.2	C4—C3—H3	119.0
C5—C6—H6	119.2	C6—C7—C2	121.8 (3)
C13—C14—C9	121.5 (2)	C6—C7—H7	119.1
C13—C14—H14	119.3	C2—C7—H7	119.1
C9—C14—H14	119.3	C3—C4—C5	120.5 (3)
C14—C13—C12	121.6 (2)	C3—C4—H4	119.8
C14—C13—H13	119.2	C5—C4—H4	119.8
C12—C13—H13	119.2	C7—C2—C3	116.8 (3)
N2—C15—H15A	109.5	C7—C2—C1	121.8 (3)
N2—C15—H15B	109.5	C3—C2—C1	121.4 (3)
H15A—C15—H15B	109.5	C10—C9—C14	116.6 (2)
N2—C15—H15C	109.5	C10—C9—C8	120.6 (2)
H15A—C15—H15C	109.5	C14—C9—C8	122.8 (2)
H15B—C15—H15C	109.5	N2—C12—C11	121.6 (2)
N2—C16—H16A	109.5	N2—C12—C13	121.4 (2)
N2—C16—H16B	109.5	C11—C12—C13	117.1 (2)
H16A—C16—H16B	109.5	C6—C5—C4	117.1 (2)
N2—C16—H16C	109.5	C6—C5—N1	117.5 (2)
H16A—C16—H16C	109.5	C4—C5—N1	125.3 (2)
H16B—C16—H16C	109.5	C12—N2—C15	121.1 (2)
C11—C10—C9	122.6 (2)	C12—N2—C16	121.1 (2)
C11—C10—H10	118.7	C15—N2—C16	117.7 (2)
C9—C10—H10	118.7	C8—N1—C5	120.6 (2)
C2—C1—H1A	109.5	C10—C11—C12	120.6 (2)
C2—C1—H1B	109.5	C10—C11—H11	119.7
H1A—C1—H1B	109.5	C12—C11—H11	119.7
C2—C1—H1C	109.5	N1—C8—C9	123.8 (2)
H1A—C1—H1C	109.5	N1—C8—H22E	118.1
H1B—C1—H1C	109.5	C9—C8—H22E	118.1
C2—C3—C4	122.0 (3)		

### Hydrogen-bond geometry (Å, º)

**Table:**

*D*—H···*A*	*D*—H	H···*A*	*D*···*A*	*D*—H···*A*
C11—H11···*Cg*1^i^	0.93	2.94	3.670 (2)	137

Symmetry code: (i) *x*, −*y*+3/2, *z*−1/2.

## References

[bb1] Altomare, A., Cascarano, G., Giacovazzo, C. & Guagliardi, A. (1993). *J. Appl. Cryst.* **26**, 343–350.

[bb2] Bruker (2004). *APEX2*, *SAINT* and *SADABS* Bruker AXS Inc., Madison, Wisconsin, USA.

[bb3] Farrugia, L. J. (2012). *J. Appl. Cryst.* **45**, 849–854.

[bb4] Fun, H.-K., Quah, C. K., Huang, C. & Yu, H. (2011). *Acta Cryst.* E**67**, o1273–o1274. 10.1107/S1600536811015327 PMC3089195 21754557

[bb5] Khalaji, A. D. & Simpson, J. (2009). *Acta Cryst.* E**65**, o553. 10.1107/S1600536809004905 PMC2968501 21582212

[bb6] Shah, S., Vyas, R. & Mehta, R. H. (1992). *J. Indian Chem. Soc.* **69**, 590–590.

[bb7] Sheldrick, G. M. (2008). *Acta Cryst.* A**64**, 112–122. 10.1107/S0108767307043930 18156677

[bb8] Spek, A. L. (2009). *Acta Cryst.* D**65**, 148–155. 10.1107/S090744490804362X PMC2631630 19171970

[bb9] Ünver, H., Karakas, A. & Elmali, A. (2004). *J. Mol. Struct.* **702**, 49–54.

[bb10] Xia, D.-G., Ye, Y.-F. & Lei, K.-W. (2009). *Acta Cryst.* E**65**, o3168. 10.1107/S1600536809044638 PMC2972087 21578884

